# Activation of HIV-1 expression in latently infected CD4+ T cells by the small molecule PKC412

**DOI:** 10.1186/s12985-016-0637-9

**Published:** 2016-10-21

**Authors:** Zhujun Ao, Rong Zhu, Xiaoli Tan, Lisa Liu, Liyu Chen, Shuiping Liu, XiaoJian Yao

**Affiliations:** 1 Department of Microbiology, School of Basic Medical Sciences, Central South University, Changsha, Hunan 410078 People’s Republic of China; 2 Laboratory of Molecular Human Retrovirology, Department of Medical Microbiology, Rady Faculty of Medicine, University of Manitoba, Winnipeg, MB Canada

**Keywords:** HIV latency, PKC412, NF-κB signaling, ACH2 cells, Resting CD4+ T cells

## Abstract

**Background:**

HIV-1 latency is a major obstacle for HIV-1 eradication. Extensive efforts are being directed toward the reactivation of latent HIV reservoirs with the aim of eliminating latently infected cells via the host immune system and/or virus-mediated cell lysis.

**Results:**

We screened over 1,500 small molecules and kinase inhibitors and found that a small molecule, PKC412 (midostaurin, a broad-spectrum kinase inhibitor), can stimulate viral transcription and expression from the HIV-1 latently infected ACH2 cell line and primary resting CD4+ T cells. PKC412 reactivated HIV-1 expression in ACH2 cells in a dose- and time-dependent manner. Our results also suggest that the nuclear factor κB (NF-κB) signaling could be one of cellular pathways activated during PKC412-mediated activation of latent HIV-1 expression. Additionally, combining PKC412 with the HDAC inhibitor vorinostat (VOR) had an additive effect on HIV-1 reactivation in both ACH2 cells and infected resting CD4+ T cells.

**Conclusions:**

These studies provide evidence that PKC412 is a new compound with the potential for optimization as a latency-reactivator to eradicate HIV-1 infection.

## Background

HIV latency has been defined as a reversibly nonproductive state of infection of individual cells that retain the capacity to produce infectious virus particles but allow the virus to evade the host immune response [1]. Several mechanisms can silence HIV gene expression and replication in HIV-1 latently infected resting CD4 + T cells, especially transcriptional interference and the chromatin environment. Other transcription factors in addition to Tat that are required for viral expression, such as nuclear factor κB (NF-κB), positive transcription elongation factor b (P-TEFb), nuclear factor of activated T cells (NFAT) and activator protein 1 (AP-1), are either insufficiently expressed or sequestered in an inactive form in resting cells [2–4]. The chromatin environment also influences the establishment and maintenance of proviral quiescence [5–9]. Chromatin remodeling enzymes such as histone deacetylases (HDACs) maintain a hypoacetylated state of local histones, which diminishes the accessibility of the nucleosomal DNA to transcription factors. In contrast, transcriptional activators such as NF-κB and NFAT, recruit histone acetyltransferases (HATs) and this results in an open or accessible DNA conformation that is more amenable to the binding of additional transcriptional activators, initiation factors, and RNA polymerase II, and leads to an active transcription [10]. In addition to HDAC activity, DNA methylation is another latency mechanism that inhibits HIV transcription [11–14].

HIV latency is the major barrier to an HIV-1 cure. Although combination antiretroviral therapy (cART) has efficiently decreased the viral load in patients, the proviral latency established within the host genome remains largely unaffected by cART and can replenish systemic infection following interruption of therapy [15, 16]. Therefore, extensive research efforts have been focused on finding ways to reactivate HIV-1 latent reservoirs and force them to be exposed to the antiretroviral therapy and the host immune system. Therapies that interfere with HDAC1 or DNA methylation are promising candidates to reactivate the suppressed virus and purge the latent HIV-1 reservoir. HDAC inhibitors (HDACIs) have received the most attention as potential latency-reversing agents. A panel of HDACIs, including trichostatin A (TSA), valproic acid (VPA), vorinostat (VOR, or suberoylanilide hydroxamic acid (SAHA)), can reactivate HIV-1 expression in latently infected cell lines, latently infected primary T cells and resting CD4+ T cells isolated from HIV-1 infected patients [17–20]. VOR has entered clinical trials to evaluate whether it can induce virus production in HIV-1-infected patients on cART [18–20]. In a 2012 pilot study, 11 of 16 patients treated with cART showed potential susceptibility to VOR [21]. Eight of these patients were administered one dose of VOR and they showed a marked increase in their HIV RNA expression levels compared with baseline. However, another study did not observe the induction of HIV production, in response to HDACIs including VOR, from latent viral reservoirs in aviremic individuals [22]. Recently, two new HDACIs (romidepsin and panobinostat) were shown to increase HIV-1 transcription and plasma RNA levels in ART-suppressed participants [23, 24]. However, although romidepsin and panobinostat effectively disrupted HIV latency in vivo, they did not affect the number of latently infected cells, suggesting that HDACIs might need to be combined with other approaches to reduce the latent HIV reservoir.

Agents associated with T cell activation through signal transduction pathways can also usually reactivate HIV latency in cell models. The protein kinase C (PKC) signaling pathway plays an important role in NFAT, NF-κB, and AP-1 activation; these steps are essential for T cell activation. PKC agonists such as phorbol esters, prostratin, bryostatin-1, and ingenol are effective at inducing viral expression from both latently infected cell lines and primary cells [25–30]. Disulfiram is a latency-reversing agent (LRA) that can reactivate latent HIV-1 in primary CD4+ T cell models without inducing global T cell activation. Disulfiram reactivates latent expression through NF-κB pathway activation [31, 32]. However, a pilot study showed that disulfiram administration to patients receiving ART did not significantly reduce the size of the latent reservoir [33, 34].

PKC412 is a derivative of the naturally occurring alkaloid staurosporine and has been shown to inhibit the conventional PKC isoforms (α, β_I_, β_II_, and γ). PKC412 targets a wide range of cell signaling pathways, including the FMS-like tyrosine kinase (FLT3) receptor, PKC, vascular endothelial growth factor receptor (VEGFR2), and c-kit (stem cell factor) receptor pathways [35, 36]. Because of its potent anti-proliferative activity, PKC412 has successfully passed a phase II clinical trial for the treatment of acute myeloid leukemia (AML) and myelodysplastic syndrome (MDS) [37–39]. Sharkey et al. found that PKC412 activity was associated with ROS generation, up-regulation of phosphorylated c-JUN, increased AP-1, and NF-κB transcription activity [40].

The present study demonstrates that PKC412 potently activates HIV-1 latency from the HIV-1 latently infected ACH2 cell line and primary resting CD4+ T cells by activating the NF-κB pathway. This study provides new insights into activators of HIV latency and it also provides compelling evidence for a novel HIV eradication strategy.

## Methods

### Reagents, antibodies, cells, and viruses

Approximately 1,500 synthesized small molecules from ChemBridge, Inc. (San Diego, USA), as described previously [41] and a Screen-well Kinase inhibitor library (Cat No. BML-2832-0100, total 80 inhibitors), obtained from Enzo Life Sciences (Farmingdale, New York, USA), were used to screen the potential HIV-1 latency reactivator. PKC412 was supplied by LC Laboratories (Woburn, USA). VOR was obtained through the NIH AIDS Reagent Program, Division of AIDS. PMA, butyrate, TNF-α, 5-aza-2’deoxycytidine (5-azac) and aphidicolin were purchased from Sigma-Aldrich, Stemcell Technologies, Abcam and Fisher Scientific, respectively. Anti-HIV-1 gp120 was obtained through NIH AIDS Reagent Program, Division of AIDS. Anti-HIV-1 p24 and was described previously [42]. Anti-β-tubulin, anti-acetyl-histone H3 and anti-histone H4, anti-NF-κB p65 (C-20) and anti-P-NF-κB p65(Ser^311^) antibodies were purchased from Santa Cruz Biotechnology (Dallas, USA). Horseradish peroxidase-conjugated donkey anti-rabbit IgG or sheep anti-mouse IgG (Amersham Biosciences, Piscataway, USA) was used as the secondary antibody.

Human embryonic kidney 293 T cells, A3.01 cells and HIV-infected ACH2 cells were cultured in Dulbecco’s modified Eagle’s medium (DMEM) or RPMI 1640 medium supplemented with 1 % or 10 % fetal bovine serum (FBS). Peripheral blood mononuclear cells (PBMCs) were isolated from the blood of healthy adult volunteers by sedimentation in Ficoll-Hypaque (Sigma-Aldrich, St. Louis, USA). CD4+ T lymphocytes were isolated from PBMCs by negative selection with the EasySep Human CD4 + CD25+ T Cell Isolation Kit (Stemcell Technologies, Vancouver, Canada).

The HIV-1 pNL4.3 virus stock was generated by transfecting 293 T cells with the corresponding HIV-1 proviral DNA, as previously described [42]. Forty-eight hours post-transfection, the supernatants were collected and subjected to ultracentrifugation (35,000 rpm for 1.5 h at 4 °C) to concentrate the viral particles.

### Stimulation of latently infected cell lines or primary infected resting CD4+ T cells

#### Screening agents that can reactivate HIV-1 latency

The synthesized small molecules and kinase inhibitors were individually dissolved in DMSO at a concentration of 5 mg/ml and stored at −20 °C until they were used. To screen the latency-reversing activity of these small molecules, each compound was added to a single well of a 96-well plate at a final concentration of 2 μM. Then, 1 × 10^4^ ACH2 cells cultured in RPMI medium supplemented with 1 % FBS for 2 days were immediately added to the 96-well plates containing the synthesized molecules. Two days later, the supernatants were collected and the HIV-associated Gag p24 levels were measured by anti-HIV p24 ELISA. DMSO-treated ACH2 cells were included as the negative control and PMA-treated ACH2 cells were included as a positive control.

#### The stimulatory effect of PKC412

ACH2 cells were cultured in RPMI medium containing 1 % FBS for 2 days and then treated with different concentrations of PKC412, VOR, or other agents for different time points. PMA (2 ng/ml) and TNF-α (10 ng/ml) were used as the positive controls and DMSO was used as the negative control. After 48 h, the cell culture supernatants were collected and HIV p24 production was measured. To examine HIV-1 expression after pulse treatment with PKC412, ACH2 cells were treated with PKC412 (1 μM) for 8, 12, 16, and 24 h and then washed. After 48 h, the cell culture supernatants were collected and HIV p24 production were measured. The cells were subjected to the immunofluorescence or Western blotting assay.

#### HIV-1-infected primary CD4+ T cell model

Unstimulated primary CD4+ T lymphocytes isolated from PBMCs were cultured in RPMI medium supplemented with 10 % FBS and IL-2 (10 U/ml) for 2 days. Cells (1 × 10^6^) were infected with 10 ng (P24 Gag) of HIV-1 pNL-4.3 via spinoculation at 1400 rpm for 2 h at room temperature. After spinoculation, the cells were washed and cultured in RPMI medium containing IL-2 for 4 days. Then, the cells were incubated with PKC412 and/or VOR at the indicated concentration for 48 h. Virus production was measured in the supernatant by detecting the levels of HIV-1 Gag p24.

### NF-κB and AP-1 activity luciferase reporter assay

VSV-G pseudotyped lentiviral particles (Cignal Lenti vector) expressing the reporter firefly luciferase gene under the control of a minimal (m)CMV promoter and tandem repeats of the AP-1 or NF-κB transcriptional response element (TRE) (QIAGEN, Hilden, Germany) were used to monitor NF-κB and AP-1 signaling activity upon treatment of the different cell lines with the compounds. Briefly, target cells were transduced with the Cignal Lenti vector. Following transduction, the cells were cultured under puromycin selection to generate a homogenous population of transduced cells. Then, the cultures were treated with PKC412 or TNF-α for 12 to 24 h and subjected to the luciferase assay [43].

### Western blotting and immunofluorescence assays

To detect various viral and cellular protein expressions, ACH2 cells were treated with PKC412 for 12–48 h, and cells were lysed with RIPA buffer and run on a 12 % SDS-PAGE gel, followed by immunoblotting with various antibodies, including anti-HIV-1 gp120, anti-HIV-1 p24, anti-tubulin, anti-acetyl-histone H3, anti-histone H3, anti-NF-κB p65, or anti-P-NF-κB p65 (Ser^311^). The protein bands were visualized using an enhanced chemiluminescence kit (Perkin Elmer Life Science, Boston, USA).

For subcellular protein fractionation and detection, a Thermo Scientific Subcellular Protein Fractionation Kit (Thermo Scientific, Waltham, USA) was used to prepare cytoplasmic, nuclear fractions from ACH2 cells, according to manufacturer’s recommended procedures. Then, each subcellular fraction was subjected to Western Blotting to detect NF-κB p65, Histone-4, β-tubulin by using specific antibodies.

For the indirect immunofluorescence assay, ACH2 cells were treated with PKC412 and then plated on a slide, fixed and permeabilized with methanol/acetone (1:1 ratio) for 30 min at room temperature. The cells were incubated with the mouse anti-p24 antibody (1:100) for 2 h at 37 °C, followed by incubation with an anti-mouse IgG-488 antibody (1:500) for 1 h. The slide was mounted with Mowiol 4–88 and the cells were visualized under an Axiovert 200 microscope (Carl Zeiss, Oberkochen, Germany).

### Cytotoxicity assay and cell cycle profiles

The trypan blue and WST-1 cell proliferation assay (Roche, Basel, switzerland) were used to determine the effect PKC412 on the cell viability and cytotoxicity, as previously described [41]. Briefly, ACH2 cells or primary CD4+ T cells were cultured at a density of 4 × 10^3^ cells/well in a 96-well format and maintained at 37 °C in the presence of various concentrations of PKC412. On day 2, cell viability was assessed using a TC20 Automated Cell Counter (Bio-Rad) or WST-1 method. The concentration of PKC412 that resulted in a 50 % decrease in cell proliferation was defined as the cell culture inhibition concentration (CCID_50_) of the compound.

The ACH2 cells were treated with PKC412 and/or aphidicolin for 24 h. The cell cycle profiles were measured by staining with propidium iodide (PI) and analyzed by flow cytometry.

### Chromatin immunoprecipitation (ChIP) assay

#### Acetylation of Histone H3 at the HIV-1 LTR promoter

ChIP assays were performed as previously described [44]. Briefly, 5 × 10^6^ ACH2 cells were mock treated or treated with PKC412, VOR or butyrate for 48 h, and crossed-linked with 1.42 % formaldehyde. The reaction was stopped with 125 mM glycine. Cells were lysed and the cross-linked chromatin was sonicated to fragment 500–1000 base pairs using a Bioruptor standard sonicator. Immunoprecipitation reaction was performed with 5 μg of anti-acetyl-histone H3 (Ac-H3 catalogue #17–615, Millipore, Darmstadt, Germany) or rabbit pre-immune immunoglobulin G (negative control). PCR of immunoprecipitates was performed using primers targeting the Nuc1 region of HIV-1LTR. The primer sequences spanning +96 to +301 nucleotides within HIV-1 LTR were the following: forward 5-AGTAGTGTGTGCCCGTCTGT-3′ and reverse 5-TTGGCGTACTCACCAGTCGC-3′. The copy of HIV-1 promoter DNA was determined by comparing the cycle threshold values of each reaction to a standard curve generated from HIV-1 provirus DNA and is reported as fold change relative to negative control.

The methylated DNA at HIV-1 LTR promoter and H19 imprinted control region (H19ICR) was detected using an EpiMark methylated DNA Enrichment kit (New England BioLabs, Ipswich, USA), according to manufacturer’s protocol. Briefly, 1 × 10^6^ ACH2 cells were mock treated or treated with PKC412, 5-aza-2′deoxycytidine (5-azac) for 48 h. The DNA in the cells were fragmented and they bound to the methyl-CpG binding domain of human MBD2 protein fused to the Fc tail of human IgG1 (MBD2-Fc), which is coupled to protein A magnetic beads. Following the magnetic capture, the enriched DNA samples were eluted and the precipitated DNA was analyzed by PCR with primers for HIV-1 LTR U3 (−300 to + −92; forward, 5′-GTTAGAGTGGAGGTTTGACAG-3′ and reverse, 5′-AGACCCAGTACAGGCAAAAAG-3′) and H19ICR (forward, 5′-ATCCCCAGCCTTTTACTGAACT-3′ and reverse, 5′-CAAACCTGCATTGAATGAG-3′). For each reaction, 10 % of the recovered DNA was used as an import control. The ChIP-qPCR results were reported as the relative fold of enrichment when comparing the ChIP fraction Ct values of input- and background- normalized experimental samples with that of the control sample.

### Measurement of intracellular HIV-1 RNA transcripts

ACH2 cells were treated with PKC412 or/and VOR for 24 to 48 h and intracellular RNA was extracted using High Pure RNA isolation (Roche) and subjected to reverse transcription with MLV reverse transcriptase (Promega, Madison, USA). HIV transcription was measured by real time PCR using primers corresponding to the HIV 5′LTR and *gag* (R-gag) (forward, 5′-ATCAAGCAGCCATGCAAATG-3′, and reverse, 5′-CTGAAGGGTACTAGTA GTTCC-3′) and normalized to the GAPDH gene levels using following primers: 5-TGGGTGTGAACCATGAGAAG-3; 5-ATGGACTGTGGTCATGAGTC-3.

### Statistical analysis

Statistical analysis was performed using GraphPad Prism version 5.0 (GraphPad Software, La Jolla, USA).

## Results

### PKC412 reactivates HIV-1 expression in latently infected ACH2 cells

**Fig. 1:**
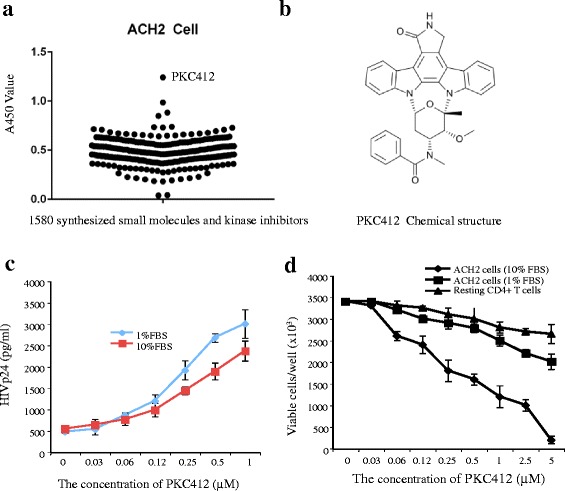
PKC412 stimulates HIV-1 expression in latently infected ACH2 cells. **a** A over 1,500 small molecules and kinase inhibitors were tested in HIV latently infected ACH2 cells in 96-well plates at a final concentration of 2 μM. After two days, the HIV-1 p24 level in each well was measured by ELISA. **b** PKC412 chemical structure. **c** ACH2 cells cultured in RPMI medium containing 1 % or 10 % FBS were treated with PKC412 at different concentrations for 48 h; then, HIV p24 production was measured in the cell culture supernatants. Error bars represent variations between duplicate samples and the data are representative of results obtained in three independent experiments. **d** Assessment of PKC412 cytotoxicity by the trypan blue dye exclusion assay. ACH2 cells in 1 % or 10 % FBS medium and human resting CD4+ T cells were treated with different PKC412 concentrations. After 48 h, the cells were assessed using the trypan blue dye exclusion assay and counted using a TC20 Automated Cell Counter. Error bars represent variation between duplicate samples and the data are representative of results obtained in three independent experiments

The HIV-1 infected ACH2 cell line, which is a subclone of a chronically infected A3.01 T lymphocyte cell line that expresses the integrated HIV-1 genome at a very low level [45, 46], was used in this study to screen reactivating agents. To isolate the potential HIV-1 latency reactivator, a 1500-synthesized small molecule library that was previously described [41], and a kinase inhibitor library were screened at a final concentration of 2 μM. The HIV-1 expression stimulated by each molecule was measured with an HIV p24 ELISA. To induce a relative quiescent state in the in vitro cellular model, proliferating ACH2 cells were cultured in serum starvation medium containing only 1 % FBS starting 48 h before treatment [47]. As shown in Fig. 1a, among the screened compounds, PKC412 (also named as RHE-12) induced significant HIV-1 production in the ACH2 cells. PKC412, 4'-N-Benzoyl-staurosporine (Fig. 1b), is an orally available staurosporine derivative that inhibits protein kinase C. This effect of PKC412 on the activation of HIV-1 production was further evaluated by treating ACH2 cells with different concentrations of compound (ranging from 1 to 0.03 μM) (Fig. 1c). The DMSO (without PKC412)-treated cells were included as control. Result showed that PKC412 upregulated virus production in a dose-dependent manner. The effect of PKC412 on the activation of HIV-1 production in the serum starved ACH2 cells was more obvious than the effect in medium supplemented with 10 % FBS. Consistent with previous studies showing that PKC412 exhibited broad anti-proliferative activity against various tumor and normal cell lines [48, 49], a proliferation inhibition effect of PKC412 was observed in proliferating ACH2 cells with a CCID_50_ of 0.4 μM (Fig. 1d and data not shown). However, the cytotoxicity of PKC412 was relatively low in the serum-starved ACH2 cells and human resting CD4+ T cells (Fig. 1d). Therefore, the highest concentrations of PKC412 used in our study were 0.5 μM in the ACH2 cells and 1 μM in the human resting CD+ T cells.

**Fig. 2:**
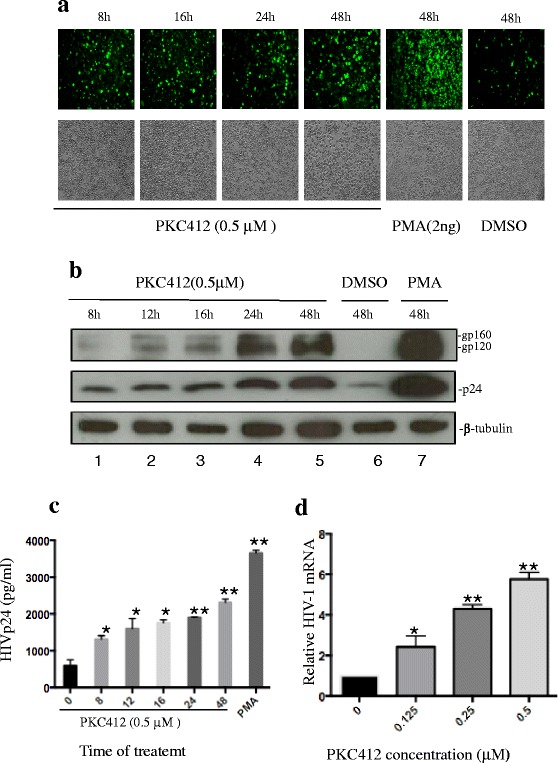
Pulse PKC412 treatment stimulates HIV-1 expression in ACH2 cells. ACH2 cells were pulse-treated with PKC412 (0.5 μM) for 8, 12, 16, 24, or 48 h. PMA (2 ng/ml) or DMSO-treated cells were used as the positive and negative controls, respectively. **a** After 24 h, the cells were fixed and labeled with an anti-HIV-1 p24 antibody/anti-mouse IgG-FITC antibody and visualized under the fluorescence microscope (10× magnification). **b** After 48 h, the cells were lysed and analyzed by SDS-PAGE followed by Western blotting with anti-HIV-1 gp120, anti-HIV-1 p24, and anti-tubulin antibodies. **c** The HIV p24 levels in supernatants were quantified using an HIV-1 p24 ELISA kit after 48 h. **d** ACH2 cells were incubated with RPMI medium (1 % FBS) containing different PKC412 concentrations. After 48 h, total RNA was extracted from the PKC412-treated or untreated ACH2 cells and HIV transcription was measured by real-time PCR using primers corresponding to the HIV 5′LTR and *gag* (R-gag). Transcription activity was calculated as the relative HIV-1 mRNA level by setting the HIV-1 mRNA level in the control ACH2 cells (without PKC412 treatment) to 1 arbitrary unit. Error bars represent variations between triplicate samples and the data are representative of results obtained in three independent experiments. The degree of significance for PKC412 treatment was relative to DMSO treatment. * *p* < 0.05, ** *p* < 0.01

We then examined whether PKC412-induced HIV-1 virus production occurred as a result of increased HIV-1 expression. A time course response experiment was performed in ACH2 cells treated with PKC412. Intracellular expression of the HIV-1 viral proteins was evaluated with anti-HIV p24 immunofluorescence and we found that the numbers of HIV Gag p24-positive cells increased in a time-dependent manner upon PKC412 treatment (Fig. 2a). The enhanced expression of HIV Gag p24, gp120, and gp160 in the ACH2 cells after PKC412 treatment was confirmed by Western blotting analysis (Fig. 2b). As expected, the increased viral protein expression levels in the cells treated with PKC412 corresponded with the augmented HIV-1 production detected in the culture supernatants (Fig. 2c), indicating that PKC412 stimulated viral protein expression.

We analyzed whether PKC412 induced HIV-1 expression at the transcription level. Briefly, the quantitative PCR technique was used to monitor the HIV-1 mRNA levels in ACH2 cells treated with or without PKC412 by measuring HIV-1 *gag* mRNA expression using primers corresponding to the HIV 5′LTR and *gag* (R-gag). As shown in Fig. 2d, PKC412 increased the *gag* mRNA levels by 1- to 6-fold at different concentrations, suggesting that PKC412 acted by inducing HIV transcription. Further evidence to support the effect of PKC412 on HIV-1 transcription was that PKC412 treatment increased HIV LTR-driven luciferase expression in the TZMb-1 cell line, which harbors an integrated HIV-1 LTR-driven luciferase gene (data not shown). No HIV proteins are expressed in TZMb-1 cells, suggesting that PKC412-mediated HIV LTR activation does not require the presence of viral proteins such as Tat.

### PKC412-induced cell cycle G2 arrest is not responsible for its HIV reactivation activity

**Fig. 3:**
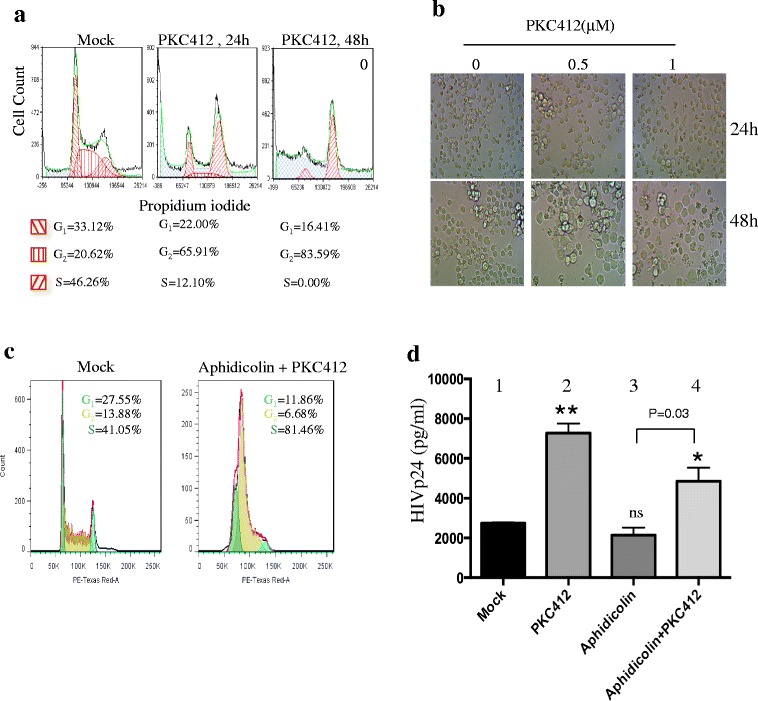
The effect of PKC412 on HIV-1 re-activation is independent of PKC412-induced cell cycle G2 arrest. **a** ACH2 cells were treated with PKC412 (0.5 μM) for 24 h and the cell cycle profiles were measured by staining with propidium iodide (PI) and analyzed with flow cytometry. **b** After 24 or 48 h, the cells were fixed and visualized under a microscope (20× magnification). **c** ACH2 cells were treated with aphidicolin (2 μM) for 12 h and then PKC412 (0.5 μM) for another 24 h. The cell cycle profiles of the ACH2 cells were analyzed with flow cytometry. The plot shows cells in G0/G1 phase (Green), S phase (yellow) and G2/M phase (blue). **d** ACH2 cells were treated with aphidicolin (2 μM) and PKC412 (0.5 μM) alone or in combination. After 48 h, the HIV-1 p24 level in each well was measured by HIV-1 p24 ELISA. Error bars represent variations between duplicate samples and the data are representative of results obtained in three independent experiments. The results were significant (* *p* < 0.05 and ** *p* < 0.01) compared to the mock treated cells

PKC412 inhibits multi-target tyrosine kinases and causes apoptosis and cell cycle arrest at the G1 or G2 phase [49–51]. Previous studies have shown that HIV-1 Vpr also is able to induce a cell cycle G2 arrest that provide a favorable environment for stimulated HIV-1 LTR-driven transcription [52–54]. Therefore, we addressed whether G1 or G2 cell cycle arrest could contribute to the effect of PKC412 on the stimulation of HIV expression in ACH2 cells. As expected, PKC412 treatment caused G2 cell cycle arrest in the ACH2 cells (Fig. 3a). A significant enlargement in the cell size and cell death were observed 24 to 48 h post-PKC412 treatment (Fig. 3b). To elucidate the functional relationship between PKC412-mediated HIV reactivation and its G2 cell cycle arrest activity, we treated the ACH2 cells with aphidicolin for 12 h to arrest the cells at the G_1_/S border [55], and PKC412 was then added for another 24 h. Cell cycle profile analysis showed that aphidicolin treatment arrested the ACH2 cells in the G1/S phase (Fig. 3c) and modestly decreased viral production (Fig. 3d, bar 1). However, PKC412 treatment with aphidicolin still increased HIV-1 p24 expression after the cells were in G1/S phase (Fig. 3d, bar 4). It should be noticed that the HIV-1 p24 level increased in the presence of both aphidicolin and PKC412 was less than that of PKC412 treated alone (Fig. 3d, bar 4 vs. bar 2). This may be partially because of a negative effect of aphidicolin on viral expression (Fig. 3d, bar 3 vs. bar 1). Nevertheless, the results indicated that the ability of PKC412 to reactivate HIV was not dependent on the cell cycle G2 arrest.

### DNA modifications are not associated with PKC412’s HIV reactivation activity

**Fig. 4:**
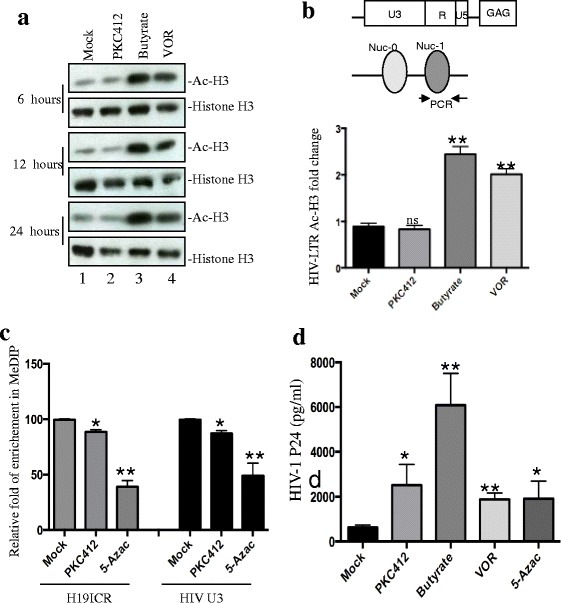
PKC412 reactivation is not associated with chromatin modulation. **a** Western blotting detection of acetylated histone H3 levels in latently infected cells. ACH2 cells were mock treated or treated with PKC412 (0.5 μM), butyrate (5 mM) or VOR (330 nM) and the cells were collected at different time points. Western blotting analysis was performed with anti-acetyl-histone H3 and anti-histone H3 antibodies. **b** The diagram shows the positions of the nucleosomes bound to the HIV-1 LTR and the location of the primers used for the real-time PCR in the ChIP assay (upper panel). Cells treated with or without PKC412 were assayed by ChIP with an anti-acetyl-histone H3 antibody. Immunoprecipitation with IgG served as the negative control. Cells treated with butyrate or VOR were used as the positive control. Real-time quantitation of the fold change relative to the negative control is shown (lower panel). **c**, **d** ACH2 cells were treated with PKC412 (0.5 μM) or the methyltransferase inhibitor 5-Azac (5 μM) for 24 h and the methylation levels of the H19 imprinted control region (ICR) and the HIV-1 LTR U3 region were detected by real-time PCR using specific primers following MeDIP ChIP with anti-5-methlcytosine (**c**). HIV-1 productions were measured with an HIV-1 p24 ELISA kit (**d**). Error bars represent variations between duplicate samples and the data are representative of results obtained in two independent experiments. The results were significant (* *p* < 0.05 and ** *p* < 0.01) compared to the mock treated cells

Previous studies showed that treatment of latently HIV-1-infected cell lines with HDACIs induced viral transcription [18–20, 56]. To test the possibility that PKC412 reactivated HIV by affecting histone acetylation, we examined the histone acetylation of ACH2 cells following PKC412 treatment by Western blotting using anti-acetyl-histone H3 and anti-histone H3 antibodies. The HDACIs VOR and butyrate were used as the positive controls. The results revealed that PKC412 did not have an effect on histone H3 acetylation (Fig. 4a line 2), whereas butyrate and VOR significantly increased the level of acetylated histone H3 (Fig. 4a, Lines 3 and 4). This result was confirmed using ChIP with anti-acetyl-histone H3 followed by real-time PCR with primers located within the nucleosome-1 (nuc-1) of the HIV-1 LTR promoter (Fig. 4b).

Because DNA methylation has been proposed as a vital transcription restriction factor that contributes to the maintenance of HIV latency and the promoter region in the 5′LTR of HIV-1 is epigenetically regulated by DNA methylation [12, 14, 57], we investigated the level of CpG methylation in the HIV-1 5’LTR after PKC412 treatment using the ChIP method using anti-methylated cytosine followed by real-time PCR with primers located in the imprinting control region (ICR) of the H19 gene and U3 of the HIV-1 5’LTR. Cells treated with the methylation inhibitor 5-azac were used as the positive control. The results showed that although 5-azac decreased the DNA methylation of H19 ICR and the HIV-1 LTR by approximately 50–60 %, PKC412 only reduced DNA methylation by approximately 10 % (Fig. 4c), which was inconsistent with the level of p24 production detected in the culture supernatant (Fig. 4d). Taken together, these observations indicate that DNA acetylation and methylation events are not responsible for the HIV LTR activating effect of PKC412.

### PKC412 induces the HIV-1 LTR and promotes gene transcription by increasing NF-κB activity in ACH2 cells

**Fig. 5:**
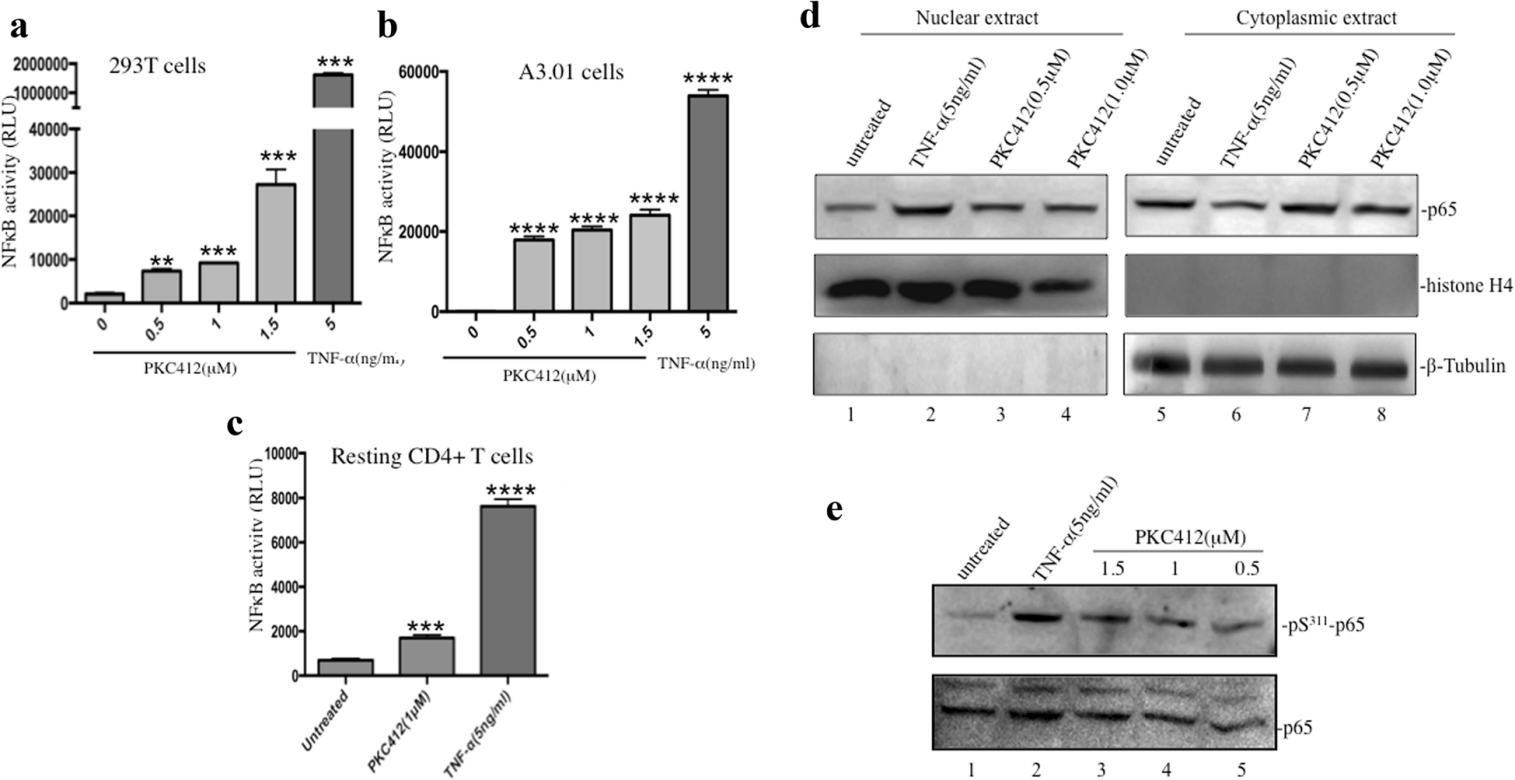
PKC-412 activates the NF-kappa B signaling pathway. The 293 T cells (**a**), A3.01 cells (**b**), and resting PBMCs (**d**) were transduced by Cignal Lenti particles encoding a luciferase (luc) gene under the control of a minimal (m)CMV promoter (Promo) and tandem repeats of the NF-κB transcriptional response element (TRE). After being transduced, cells were treated with different concentrations of PKC412 and TNF-α for 12 h and activation of NF-κB signaling was detected by measuring the luc activity. **d** ACH2 cells were treated with various concentrations of PKC412 and TNF-α for 12 h, and then cells were fractionated into nuclear and cytoplasmic fractions. The nucleus- and cytoplasm-associated p65, histone-4 and β-tubulin were detected by WB with corresponding antibodies. **e** ACH2 cells were treated with various concentrations of PKC412 and TNF-α for 12 h. Then the cells were lysed, and Ser^311^phosphorylated p65 was detected by WB with corresponding antibody. Error bars represent variations between triplicate samples and the data are representative of results obtained in three independent experiments. The degree of significance for the PKC412 or TNF-α treatment was reported relative to the mock treatment. ** *p* < 0.01, *** *p* < 0.001, and **** *p* < 0.0001

PKC412 was previously reported to upregulate c-JUN phosphorylation and NF-κB and AP-1 transcription activity in human multiple myeloma cells [40]. Thus, we tested whether PKC412 could activate the NF-κB or AP-1 signaling pathways by using NF-κB- or AP-1-Cignal Lenti luciferase report assay. After transducing 293 T and A3.01 cells (the parental CD4+ T cell line of ACH2 cells) with NF-κB-Cignal Lenti particles, the effect of the various treatments on the activity of the NF-κB or AP-1 signaling pathways can be easily detected by measuring the luc activity in transduced cells, as described in the Materials and Methods. The TNF-α treated cells were included as positive control. The results revealed that PKC412 treatment significantly activated the NF-κB activity in a dose-dependent manner in both the 293 T and A3.01 cells (Fig. 5a and b), whereas both VOR and 5-azac only showed a modest stimulating effect on the NF-κB pathway (Fig. 5a and data not shown). In contrast, PKC412 treatment did not show any stimulating effect the AP-1 signal pathway in, A3.01 cells, the parental cell lines of ACH2 (data not shown). We further tested the NF-κB-Cignal Lenti luciferase report assay in human resting CD4 + T cells, and the results showed that PKC412 induced approximately 2–3-fold higher luc activity than the mock transduced cells, whereas TNF–α treatment induced 10-fold higher luc activity (Fig. 5c). These data suggest that NF-κB signing may be involved in the HIV reactivating activity of PKC412.

To further assess how PKC-412 is able to activate NF-κB signaling, we first investigated the NK-κB nuclear translocation by detecting nucleus- and cytoplasm-associated p65 levels after ACH2 cells being treated by PKC-412. PKC-412 treatment only resulted in a modest increase of nucleus-associated NF-κB p65, compared to no treatment (Fig. 5d, lanes 3–4 to lane 1). Since it is known that NF-κB phosphorylation at position of Ser311 plays an important role in enhancing NF-κB-mediated gene transcription [58, 59], we also assessed the NF-κB Ser311 phosphorylation level at PKC-412 treated ACH2 cells. Interestingly, there was a significant increase in Ser311 phosphorylation of NF-κB p65 in ACH2 cells following PKC-412 treatment (Fig. 5e, compare lanes 3–5 to lane 1), indicating that PKC-412 treatment facilitates Ser311 phosphorylation of NF-κB, which may contribute to its increased transcription signaling in ACH2 cells.

### Additive effect of PKC412 and the HDACI VOR on the activation of HIV-1 expression in ACH2 cells and primary resting HIV-infected CD4+ T cells

**Fig. 6:**
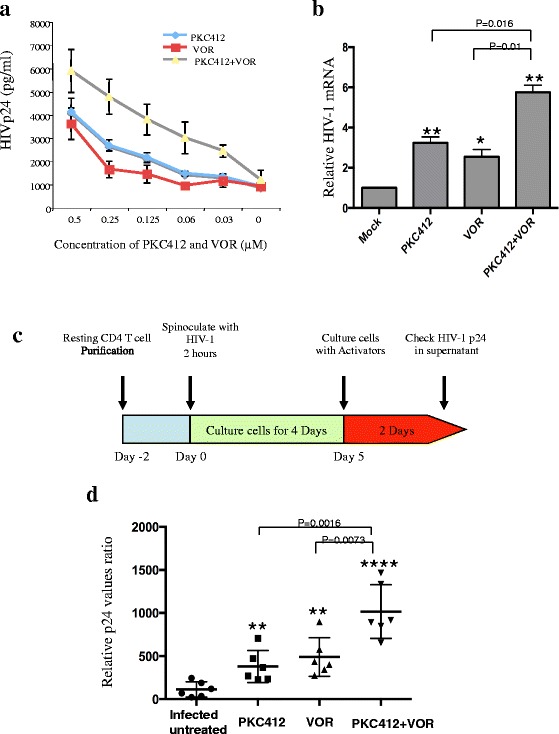
Additive effect of PKC412 and VOR on the activation of latent HIV-1 expression. **a** ACH2 cells were treated with different concentrations of PKC412 and/or VOR. After 48 h, the activating effect of PKC412 and/or VOR on HIV expression was determined by measuring the HIV p24 level in the supernatants. **b** HIV transcript levels in ACH2 cells treated with PKC412 and/or VOR were measured with real-time PCR using primers corresponding to the HIV 5′LTR and *gag* (R-gag) and calculated as relative HIV-1 mRNA levels to non-treated ACH2 cells. **c** The schematic diagram of HIV infection in resting CD4+ T cells and the effects of PKC412 and/or VOR. **d** The infected resting CD4+ T cells from different donors were treated with PKC412 and/or VOR or untreated for 2 days. Then, the HIV Gag p24 levels from the supernatants were monitored by ELISA and calculated as relative P24 levels compared to the infected-untreated cells. The experiments were performed as triplicate repeats per assay for each donor. The mean values from each donor are shown with the standard error of the mean (SEM). The degree of significance for PKC412 or VOR treatment was relative to the infected-untreated cells. * *p* < 0.05, ** *p* < 0.01, *** *p* < 0.001, and **** *p* < 0.0001

Multiple mechanisms contribute to the maintenance of proviral latency. Therefore, the use of combination latency reversal strategies might optimize the reactivation of silenced proviral DNA. The results described above indicate that PKC412 induced NF-κB signaling but not DNA modifications, whereas VOR primarily affected the level of acetylated histones. Therefore, we tested whether PKC412 acted synergistically with VOR on HIV expression. As expected, we observed a dose-dependent proviral response following increased exposure of up to 0.5 μM VOR or PKC412 in the ACH2 cells. Higher levels of virus production were observed when PKC412 and VOR were simultaneously added to the cell cultures (Fig. 6a). The HIV-1 transcript levels in the PKC412- and VOR-treated ACH2 cells were consistently increased, with the PKC412/VOR co-treated cells showing the highest level of HIV-1 transcription (Fig. 6b). Overall, the above results indicate that the combined use of PKC412 and VOR additively activates HIV-1 production in the ACH2 cells.

To investigate whether PKC412 can activate virus expression from naturally HIV-1-infected resting CD4+ T cells, we established an HIV-1-infected primary resting CD4+ T cell model, as shown in Fig. 6c. The resting CD4+ T cells from the blood of 6 healthy donors was infected with HIV-1 for 4 days and then exposed to PKC412 (1 μM), VOR (1 μM), or PKC412 (1 μM) + VOR (1 μM) for 48 h. The supernatants were collected to measure the HIV p24 levels. The results showed that both VOR and PKC412 alone resulted in higher levels of viral expression than non-treated HIV-1-infected resting primary cell (*p* < 0.01; (Fig. 6d). PKC412 plus VOR treatment induced the highest levels (3.6-fold) of HIV p24 antigen production compared to the non-treated cells (*p* < 0.0001; Fig. 6d). These results indicate that PKC412 is able to stimulate HIV expression in HIV-1-infected resting CD4+ T cells. Additionally, the PKC412 + VOR combined treatment can additively activate virus expression compared with PKC412 or VOR treatment alone (*p* < 0.01).

## Discussion

Recently, many studies have focused on the development of molecules that can reactivate HIV-1 latent reservoirs to render them susceptible to antiretroviral therapy, viral cytopathic effects, and host immune responses. To date, the candidate molecules under evaluation include HDAC inhibitors (HDACIs), PKC agonists, agents that induce the JNK or Akt pathway, and DNA methylation inhibitors [18, 28, 31, 32, 56, 60–64]. Because multiple mechanisms contribute to the maintenance of proviral latency, a combined therapy targeting multiple pathways would be the most effective for the activation of HIV latency. Thus, it is important to identify new agents that can activate HIV latency through different pathways. In this report, we found that the PKC412, a broad-spectrum kinase inhibitor with multiple known and unknown cellular targets, was able to reactivate viral transcription from an HIV-1 latently infected ACH2 cell line and HIV-infected primary resting CD4+ T cells. Our data also suggested that PKC412 mediated the activation of HIV-1 through its effect on the NF-κB Ser311 phosphorylation and signaling. Moreover, an additive effect on HIV-1 reactivation was observed when PKC412 was combined with the HDAC inhibitor VOR in both the ACH2 cells and primary resting CD4+ T cells.

Previous studies indicated that some transcription factors, including NF-κB, P-TEFb, NFAT, and AP-1, were either insufficiently expressed or sequestered in an inactive form in HIV-1-infected resting CD4 + T cells [2–4]. It is conceivable that activation of the NF-κB or AP-1 signaling pathway could lead to activation of HIV expression even when the cells are in a quiescent non-dividing state. Because PKC412 was previously reported to up-regulate phosphorylation of c-JUN, increased AP-1 and NF-κB transcription activities in human multiple myeloma cells [40], we tested the possibility that PKC412 stimulates HIV transcription in HIV latent infected cells through activating NF-κB or AP-1 signal pathways. Our analysis showed that PKC412 was able to up-regulate NF-κB signaling, but not AP-1, and activated HIV-1 production in latently infected ACH2 cells and human primary resting CD4+ T cells. It is known that NF-κB plays a major role in HIV-1 transcription by binding to κB sites within the HIV-1 long terminal repeat (LTR) enhancer region [65]. Generally, NF-κB is found in the cytoplasm in resting human CD4+ T cells associated with its inhibitor (IκBα), which masks the NF-κB nuclear localization signal. Some cellular stimuli trigger the phosphorylation of specific serine residues of the IκBα protein and result in the degradation of IκBα and the release of NF-κB to translocate into the nucleus and induce the transcription of many genes by binding to specific consensus sequences in their promoter regions. In addition, it is known that release from IκB may not be insufficient to allow full activation of NF-κB. Several posttranslational modifications, including phosphorylation of Ser^311^, in NF-κB after IκB degradation appear to regulate DNA binding and transcriptional transactivation [66, 67]. Our data reveals that PKC412 can significantly increase Ser^311^ phosphorylation level of NF-κB (Fig. 5e). Several studies have indicated that Ser^311^ phosphorylation can enhance NF-κB-mediated gene transcription without affecting nuclear localization [58]. Previous studies showed that an amino acid Lys310 in NF-κB could either be acetylated or methylated, which in turn up- or down-regulates NF-κB’s transactivation activity [68, 69]. Because Lys310 is in close proximity to Ser311, it has been speculated that the Ser^311^ phosphorylation may play a regulatory role for either acetylation or methylation of Lys310, which in turn optimizes or suppresses NF-κB transactivation activity [59]. PKC412-induced increased Ser^311^ phosphorylation may be one of the mechanisms that activates this effect on HIV transcription. In addition, a previous report also showed that PKC412 treatment leads to a significant elevation of HSP90 [40], which has been shown to play an important role in controlling HIV-1 reactivation from latency [70, 71]. More detailed studies will be required to elucidate the functional association of PKC-412-mediated NF-κB Ser^311^phosphorylation and increased HSP90 expression and their roles in HIV activation.

In the ACH2 cell line, both VOR and PKC412 could increase the p24 level by approximately 4.5-fold, (Fig. 6a). However, in human resting CD4+ T cells, the capacity of PKC412 to induce viral gene expression was a little lower than that of VOR (Fig. 6d). VOR is an extensively studied HDAC inhibitor that induces HIV transcription in latently infected cell lines and resting CD4+ T cells from HIV-infected patients on suppressive cART [18, 21]. However, a recent study found that the levels of HIV production stimulated from resting CD4+ T cells from aviremic donors by VOR were not significantly different from those of control treated cells [22]. A previous study demonstrated a strong synergistic activation of HIV-1 production by clinically used HDACIs including VOR combined with the non-tumor-promoting NF-κB inducer prostratin in latently infected cell lines and resting CD4+ T cells isolated from cART-treated patients [72]. Therefore, we tested the effect of combining VOR and PKC412 on viral transcription and expression and detected an additive effect in ACH2 cells (Fig. 6a and b). We also extended our studies to resting CD4+ T cells, which are the primary reservoir of HIV-1, and documented that PKC412 or VOR enhanced HIV p24 production by 1.8–2.2-fold, whereas the PKC412 + VOR combined treatment resulted in an approximate increase of p24 production of 3.6-fold. Our study did not observe a stimulating effect of PKC412 on HIV production in proliferating CD4+ T cells (data not shown), suggesting a possible specific effect on HIV expression activation in resting T cells. More studies are needed to investigate PKC412’s latent-reactivating effects alone or in combination with VOR or other latent reactive agents in resting CD4+ T cells isolated from HIV-1 infected individuals on ART and to evaluate the feasibility of developing this agent for HIV eradication.

## Conclusions

In this study, we demonstrated that the small molecule PKC412 (a broad-spectrum kinase inhibitor), induced viral transcription from latently HIV-1-infected ACH2 cells and primary resting CD4+ T cells by increasing NF-κB signaling. The combination of PKC412 with the HDAC inhibitor VOR had an additive effect on HIV-1 reactivation, suggesting that PKC412 has the potential for optimization as a latency-reactivator for the eradication of HIV-1 infection.

## Abbreviations

NF-κB: Nuclear factor κB
VOR: Vorinostat
cART: Combination antiretroviral therapy
HDACs: Histone deacetylases
HDACIs: Histone deacetylases inhibitor
PKC: Protein kinase C
P-TEFb: Positive transcription elongation factor b
NFAT: Nuclear factor of activated T cells
AP-1: Activator protein 1
ChIP: Chromatin immunoprecipitation
5-azac: 5-aza-2’deoxycytidine
